# Stress Echo 2030: The Novel ABCDE-(FGLPR) Protocol to Define the Future of Imaging

**DOI:** 10.3390/jcm10163641

**Published:** 2021-08-17

**Authors:** Eugenio Picano, Quirino Ciampi, Lauro Cortigiani, Adelaide M. Arruda-Olson, Clarissa Borguezan-Daros, José Luis de Castro e Silva Pretto, Rosangela Cocchia, Eduardo Bossone, Elisa Merli, Garvan C. Kane, Albert Varga, Gergely Agoston, Maria Chiara Scali, Doralisa Morrone, Iana Simova, Martina Samardjieva, Alla Boshchenko, Tamara Ryabova, Alexander Vrublevsky, Attila Palinkas, Eszter D. Palinkas, Robert Sepp, Marco A. R. Torres, Hector R. Villarraga, Tamara Kovačević Preradović, Rodolfo Citro, Miguel Amor, Hugo Mosto, Michael Salamè, Paul Leeson, Cristina Mangia, Nicola Gaibazzi, Domenico Tuttolomondo, Costantina Prota, Jesus Peteiro, Caroline M. Van De Heyning, Antonello D’Andrea, Fausto Rigo, Aleksandra Nikolic, Miodrag Ostojic, Jorge Lowenstein, Rosina Arbucci, Diego M. Lowenstein Haber, Pablo M. Merlo, Karina Wierzbowska-Drabik, Jaroslaw D. Kasprzak, Maciej Haberka, Ana Cristina Camarozano, Nithima Ratanasit, Fabio Mori, Maria Grazia D’Alfonso, Luigi Tassetti, Alessandra Milazzo, Iacopo Olivotto, Alberto Marchi, Hugo Rodriguez-Zanella, Angela Zagatina, Ratnasari Padang, Milica Dekleva, Ana Djordievic-Dikic, Nikola Boskovic, Milorad Tesic, Vojislav Giga, Branko Beleslin, Giovanni Di Salvo, Valentina Lorenzoni, Matteo Cameli, Giulia Elena Mandoli, Tonino Bombardini, Pio Caso, Jelena Celutkiene, Andrea Barbieri, Giovanni Benfari, Ylenia Bartolacelli, Alessandro Malagoli, Francesca Bursi, Francesca Mantovani, Bruno Villari, Antonello Russo, Michele De Nes, Clara Carpeggiani, Ines Monte, Federica Re, Carlos Cotrim, Giuseppe Bilardo, Ariel K. Saad, Arnas Karuzas, Dovydas Matuliauskas, Paolo Colonna, Francesco Antonini-Canterin, Mauro Pepi, Patricia A. Pellikka, The Stress Echo 2030 Study Group of the Italian Society of Echocardiography and Cardiovascular Imaging (SIECVI)

**Affiliations:** 1 CNR, Biomedicine Department, Institute of Clinical Physiology, 56100 Pisa, Italy; denesm@ifc.cnr.it (M.D.N.); claracarpeggiani@gmail.com (C.C.); 2 Cardiology Division, Fatebenefratelli Hospital, 82100 Benevento, Italy; qciampi@gmail.com (Q.C.); villari.bruno@gmail.com (B.V.); 3 Cardiology Department, San Luca Hospital, 55100 Lucca, Italy; lacortig@tin.it; 4 Department of Cardiovascular Medicine, Mayo Clinic, Rochester, MN 55905, USA; ArrudaOlson.Adelaide@mayo.edu (A.M.A.-O.); kane.garvan@mayo.edu (G.C.K.); Villarraga.Hector@mayo.edu (H.R.V.); Padang.Ratnasari@mayo.edu (R.P.); pellikka.patricia@mayo.edu (P.A.P.); 5 Cardiology Division, Hospital San José, Criciuma 88801-250, Brazil; clarissabdaros@cardiol.br; 6 Hospital Sao Vicente de Paulo e Hospital de Cidade, Passo Fundo 99010-080, Brazil; jlpretto@cardiol.br; 7 Hospital de Cidade, Passo Fundo 99010-080, Brazil; 8 Azienda Ospedaliera Rilevanza Nazionale A. Cardarelli Hospital, 80100 Naples, Italy; rosangelacocchia@hotmail.com (R.C.); ebossone@hotmail.com (E.B.); 9 Department of Cardiology, Ospedale per gli Infermi, Faenza, 48100 Ravenna, Italy; elisamerli@libero.it; 10 Institute of Family Medicine, Szeged University Medical School, University of Szeged, 6720 Szeged, Hungary; varga.albert@med.u-szeged.hu (A.V.); drgergoagoston@gmail.com (G.A.); 11 Campostaggia Cardiology Division, Montepulciano, 53045 Siena, Italy; chiara_scali@yahoo.it; 12 Cardiothoracic Department, University of Pisa, 56100 Pisa, Italy; doralisamorrone@gmail.com; 13 Heart and Brain Center of Excellence, Cardiology Department, University Hospital, Medical University, 5800 Pleven, Bulgaria; ianasimova@gmail.com (I.S.); martina_vl@abv.bg (M.S.); 14 Cardiology Research Institute, Tomsk National Research Medical Centre of the Russian Academy of Sciences, 634009 Tomsk, Russia; allabosh@mail.ru (A.B.); rtr@cardio-tomsk.ru (T.R.); avr@cardio-tomsk.ru (A.V.); 15 Internal Medicine Department, Elisabeth Hospital, 6800 Hódmezővásárhely, Hungary; palinkasa@hotmail.com; 16 Albert Szent-Gyorgyi Clinical Center, Department of Internal Medicine, Division of Non-Invasive Cardiology, University Hospital, 6725 Szeged, Hungary; sepprobert@gmail.com (R.S.); palinkaseszti@hotmail.com (E.D.P.); 17 9th July Hospital, DASA, San Paolo 04122-000, Brazil; mtorres.mt10@gmail.com; 18 Clinic of Cardiovascular Diseases, University Clinical Centre of the Republic of Srpska, 78 000 Banja Luka, Bosnia and Herzegovina; tamara.kovacevic@medicolaser.info (T.K.P.); tbombardini@yahoo.it (T.B.); 19 Cardiology Department and Echocardiography Lab, University Hospital “San Giovanni di Dio e Ruggi d’Aragona”, 84100 Salerno, Italy; rodolfocitro@gmail.com; 20 Cardiology Department, Ramos Mejia Hospital, Buenos Aires C1221, Argentina; miguelamor68@gmail.com (M.A.); hmosto@gmail.com (H.M.); michael.f.salame@gmail.com (M.S.); 21 RDM Division of Cardiovascular Medicine, Cardiovascular Clinical Research Facility, University of Oxford, Oxford OX3 9DU, UK; paul.leeson@cardiov.ox.ac.uk; 22 CNR, ISAC-Institute of Sciences of Atmosphere and Climate, 73100 Lecce, Italy; c.mangia@isac.cnr.it; 23 Cardiology Department, Parma University Hospital, 43100 Parma, Italy; ngaibazzi@gmail.com (N.G.); d.tuttolomondo@hotmail.it (D.T.); 24 Cardiology Department, Vallo della Lucania Hospital, 84100 Salerno, Italy; costantinaprota@gmail.com; 25 CHUAC-Complexo Hospitalario Universitario A Coruna, CIBER-CV, University of A Coruna, 15070 La Coruna, Spain; Jesus.Peteiro.Vazquez@sergas.es; 26 Department of Cardiology, Antwerp University Hospital, 2650 Edegem, Belgium; carovdh@msn.com; 27 UOC Cardiologia/UTIC/Emodinamica, PO Umberto I, Nocera Inferiore (ASL Salerno)—Università Luigi Vanvitelli della Campania, 84014 Salerno, Italy; antonellodandrea@libero.it (A.D.); pio.caso@tin.it (P.C.); 28 Department of Cardiology, Dolo Hospital, 30031 Venice, Italy; faustorigo@alice.it; 29 Department of Noninvasive Cardiology, Institute for Cardiovascular Diseases Dedinje, School of Medicine, Belgrade 11000, Serbia; nikolicdrsasa@gmail.com (A.N.); mostojic2011@gmail.com (M.O.); 30 Cardiodiagnosticos, Investigaciones Medicas Center, Buenos Aires C1082, Argentina; lowensteinjorge@hotmail.com (J.L.); rosinaarbucci@hotmail.com (R.A.); lowediego@hotmail.com (D.M.L.H.); pablommerlo@gmail.com (P.M.M.); 31 Department of Cardiology, Bieganski Hospital, Medical University, 91-347 Lodz, Poland; wierzbowska@ptkardio.pl (K.W.-D.); kasprzak@ptkardio.pl (J.D.K.); 32 Department of Cardiology, SHS, Medical University of Silesia, 40-752 Katowice, Poland; mhaberka@op.pl; 33 Medicine Department, Hospital de Clinicas UFPR, Federal University of Paranà, Curitiba 80000-000, Brazil; a.camarozano@yahoo.com.br; 34 Department of Medicine, Division of Cardiology, Siriraj Hospital, Mahidol University, Bangkok 10700, Thailand; nithimac@hotmail.com; 35 SOD Diagnostica Cardiovascolare, DAI Cardio-Toraco-Vascolare, Azienda Ospedaliera-Universitaria Careggi, 50139 Firenze, Italy; morif@aou-careggi.toscana.it (F.M.); mariagrazia.dalfonso@gmail.com (M.G.D.); luigi.tassetti.1990@gmail.com (L.T.); ales.milazzo@gmail.com (A.M.); iacopo.olivotto@unifi.it (I.O.); alb.marchi@yahoo.com (A.M.); 36 Instituto Nacional de Cardiologia Ignacio Chavez, Mexico City 14080, Mexico; drzanella@gmail.com; 37 Cardiology Department, Saint Petersburg State University Hospital, 199034 Saint Petersburg, Russia; zag_angel@yahoo.com; 38 Clinical Cardiology Department, Clinical Hospital Zvezdara, Medical School, University of Belgrade, Belgrade 11000, Serbia; dekleva.milica@gmail.com; 39 University Clinical Centre of Serbia, Medical School, Cardiology Clinic, University of Belgrade, 11000 Belgrade, Serbia; skali.ana7@gmail.com (A.D.-D.); belkan87@gmail.com (N.B.); misa.tesic@gmail.com (M.T.); voja2011@yahoo.com (V.G.); branko.beleslin@gmail.com (B.B.); 40 Division of Pediatric Cardiology, University Hospital, 35100 Padua, Italy; giodisal@yahoo.it; 41 Institute of Management, Scuola Superiore Sant’Anna, 56100 Pisa, Italy; v.lorenzoni@sssup.it; 42 Division of Cardiology, University Hospital, 53100 Siena, Italy; matteo.cameli@yahoo.com (M.C.); giulia_elena@hotmail.it (G.E.M.); 43 Centre of Cardiology and Angiology, Clinic of Cardiac and Vascular Diseases, Faculty of Medicine, Institute of Clinical Medicine, Vilnius University, LT-03101 Vilnius, Lithuania; Jelena.Celutkiene@santa.lt; 44 Noninvasive Cardiology, University Hospital, 43100 Parma, Italy; olmoberg@libero.it; 45 Cardiology Department, University of Verona, 37121 Verona, Italy; giovanni.benfari@gmail.com; 46 Paediatric Cardiology and Adult Congenital Heart Disease Unit, S. Orsola-Malpighi Hospital, 40100 Bologna, Italy; ylenia.bartolacelli@gmail.com; 47 Nephro-Cardiovascular Department, Division of Cardiology, Baggiovara Hospital, University of Modena and Reggio Emilia, 41126 Modena, Italy; ale.malagoli@gmail.com; 48 ASST Santi Paolo e Carlo, Presidio Ospedale San Paolo, 20100 Milano, Italy; francescabursi@gmail.com; 49 Azienda Unità Sanitaria Locale—IRCCS di Reggio Emilia, Cardiology, 42100 Reggio Emilia, Italy; francy_manto@hotmail.com; 50 Association for Public Health “Salute Pubblica”, 72100 Brindisi, Italy; antonellorusso72@hotmail.com; 51 Echocardiography Laboratory, Cardio-Thorax-Vascular Department, “ Policlinico Vittorio Emanuele”, Catania University, 95100 Catania, Italy; inemonte@gmail.com; 52 Ospedale San Camillo, Cardiology Division, 00100 Rome, Italy; re.federica77@gmail.com; 53 Heart Center, Hospital da Cruz Vermelha, Lisbon, and Medical School of University of Algarve, 1549-008 Lisbon, Portugal; carlosadcotrim@hotmail.com; 54 UOC di Cardiologia, ULSS1 DOLOMITI, Presidio Ospedaliero di Feltre, 32032 Belluno, Italy; bilardogiuseppe@gmail.com; 55 División de Cardiología, Hospital de Clínicas José de San Martín, Buenos Aires C1120, Argentina; arielsaad@gmail.com; 56 Ligence Medical Solutions, 49206 Vilnius, Lithuania; a.karuzas@ligence.io (A.K.); d.matuliaskas@ligence.io (D.M.); 57 Cardiology Hospital, Policlinico University Hospital of Bari, 70100 Bari, Italy; colonna@tiscali.it; 58 Italian Society of Echocardiography and Cardiovascular Imaging, 20138 Milan, Italy; antonini.canterin@gmail.com (F.A.-C.); Mauro.Pepi@cardiologicomonzino.it (M.P.); 59 Cardiac Prevention and Rehabilitation Unit, Highly Specialized Rehabilitation Hospital Motta di Livenza, Motta di Livenza, 31045 Treviso, Italy; 60 Centro Cardiologico Monzino, IRCCS, 20138 Milan, Italy

**Keywords:** effectiveness, registry, stress echocardiography, sustainability

## Abstract

With stress echo (SE) 2020 study, a new standard of practice in stress imaging was developed and disseminated: the ABCDE protocol for functional testing within and beyond CAD. ABCDE protocol was the fruit of SE 2020, and is the seed of SE 2030, which is articulated in 12 projects: 1-SE in coronary artery disease (SECAD); 2-SE in diastolic heart failure (SEDIA); 3-SE in hypertrophic cardiomyopathy (SEHCA); 4-SE post-chest radiotherapy and chemotherapy (SERA); 5-Artificial intelligence SE evaluation (AI-SEE); 6-Environmental stress echocardiography and air pollution (ESTER); 7-SE in repaired Tetralogy of Fallot (SETOF); 8-SE in post-COVID-19 (SECOV); 9: Recovery by stress echo of conventionally unfit donor good hearts (RESURGE); 10-SE for mitral ischemic regurgitation (SEMIR); 11-SE in valvular heart disease (SEVA); 12-SE for coronary vasospasm (SESPASM). The study aims to recruit in the next 5 years (2021–2025) ≥10,000 patients followed for ≥5 years (up to 2030) from ≥20 quality-controlled laboratories from ≥10 countries. In this COVID-19 era of sustainable health care delivery, SE2030 will provide the evidence to finally recommend SE as the optimal and versatile imaging modality for functional testing anywhere, any time, and in any patient.

## 1. Introduction

Stress echo (SE) 2020 is an international, multicenter, prospective, effectiveness study started in 2016 that conceptualized, disseminated, and validated a new approach for functional testing within and beyond coronary artery disease (CAD). As originally planned, the study created the cultural, informatic, and scientific infrastructure connecting high-volume, accredited SE labs, sharing common criteria of indication, execution, reporting, and archiving SE. This approach allowed acquisition of original safety, feasibility, and outcome data in evidence-poor diagnostic fields, beyond the established core application of SE in CAD based on regional wall motion abnormality (RWMA) assessment. SE2020 standardized procedures, validated emerging signs, and integrated new information with established knowledge, helping to build a next-generation SE lab adopting the ABCDE protocol [1]. Each and every step of ABCDE-SE provides independent and incremental prognostic information building on the prior steps and identifies distinct patient phenotypes and vulnerabilities possibly outlining different therapeutic targets: myocardial ischemia in step A, pulmonary congestion with B-lines in step B, preload reserve and left ventricular contractile reserve (LVCR) in step C, coronary microcirculation with coronary flow velocity reserve (CFVR) or real-time myocardial contrast echocardiography in step D, and cardiac autonomic balance with heart rate reserve (HRR) in step E [2]. This shared practice can now be used as a new standard of care [3] and a suitable platform for the next wave of studies converging towards SE 2030—sharing with the older SE2020 study some distinct features: effectiveness study, performed in the real world with real doctors facing real clinical problems in real consecutive patients; upstream quality control of reading and direct entering of data from peripheral centers in the data bank so that evidence is obtained inside and outside highly specialized academic centers; identification of simple yet innovative objectives relevant to change the clinical practice. These features are completely different from efficacy studies, as when highly specialized centers recruit highly selected patients, the resulting data may be difficult to translate in clinical practice. For these reasons, the American Society of Echocardiography has identified already in 2013 as a top research need “the development of a registry of echocardiographic information (and eventually images) that can serve as a platform for quality improvement and clinical research. Such registry data would be accessible to the research community facilitating a broad range of clinical research on the effectiveness of echocardiography for the improvement of patient management and outcome” [4].

SE2030 will establish the platform of evidence to build the perfect SE test, suitable for all patients, anywhere, anytime, that is also quantitative and operator independent. The need for such an ideal test is especially vital in our times, when economic crises, the increased awareness of cancer and non-cancer radiation damage, the pressing need for climate-neutral choices in health care, and the unavoidable trend to externalize health care are potent propelling forces, boosted by COVID pandemics, for the diffusion of a low cost, radiation-free, climate-friendly, and portable technique such as cardiovascular ultrasound [5].

## 2. Materials and Methods

Five important aspects will be shared by SE2030 in full continuity with SE2020, with minor adaptations and implementations.

### 2.1. Upstream Quality Control

The study is a prospective registry, but it is necessary to have an upstream quality control with a certified reader from each center. Peripheral reading from each center is necessary for the effectiveness study to provide a snapshot of the real world. On the other side, a multicenter study is like a fish soup. The more the fish or contributing centers, the tastier the soup, but a single rotten fish will render the entire soup uneatable. Therefore, a mandatory quality control is necessary for conventional and innovative parameters, since the volume of activity is necessary but not sufficient to ensure the quality of reading [6].

### 2.2. Peripheral Reading and Inclusivity

Once the reader has been certified, the peripheral reading will be directly entered in the data bank via the Redcap program property of the Italian Society of Echocardiography and Cardiovascular Imaging. This will allow a more flexible and rapid platform compared to the standard excel approach, which requires greater human resources and has a greater chance of error in data inputting. In addition, Redcap has better compliance with new regulations strictly protecting privacy in clinical studies. Another feature of SE2030 is inclusivity, so that any center meeting the selection criteria can be enrolled, allowing centers traditionally outside the editorial stage but producing high quality clinical activity to contribute to generate data relevant for the scientific community. The inclusion of centers from many sites contains potential to recognize similarities and differences between countries, continents, cultures, and ethnicities. Inclusivity will also allow to assess how the proposed protocol will materialize in different scenarios (private practice settings, public hospitals, academic institutions) with different reimbursement policies and variable direct costs and commercial availability for drugs (such as dipyridamole or adenosine) and ultrasound-enhancing agents.

### 2.3. Uniform Methodology

Each laboratory will adopt the preferred choice of stress among physical, pharmacologic, or pacing stress according to standardized protocol in line with guideline recommendations. Physical exercise includes semi-supine or upright bicycle exercise, and peak or post-treadmill exercise. Pharmacologic testing will be with dobutamine or vasodilators (dipyridamole, adenosine, or regadenoson) according to physician preferences, patients’ contraindications, local availability, and cost. Pacing stress can be performed with transesophageal atrial pacing or with external programming of a permanent pacemaker. Independent of the chosen form of stress, execution, performance, archiving, and interpretation of testing will follow a standardized format with the ABCDE protocol. From the technical viewpoint of success rate, a limiting step is step D. Step D is easy and feasible with vasodilator, less easy but still highly feasible with dobutamine, not easy and less feasible with semi-supine exercise, and virtually impossible with (peak or post) treadmill exercise.

Therefore, our recommendation is to use semi-supine exercise, capturing coronary flow signal in early or intermediate stages of exercise when most flow increase occurs and feasibility is still high, before it drops at higher levels of exercise. When treadmill is used, step D is skipped; if information is deemed important, a vasodilator test can be performed at 30′ after the end of exercise focused on CFVR and heart rate response.

All laboratories will be granted with free artificial intelligence (AI) software and encouraged to use ultrasound enhancing agents when needed to help leading edge technology upgrade and uniformity of methods across all study laboratories [7].

### 2.4. The Full Spectrum of Enrolled Patients Evaluated for Clinically Relevant Endpoints

**Figure 1:**
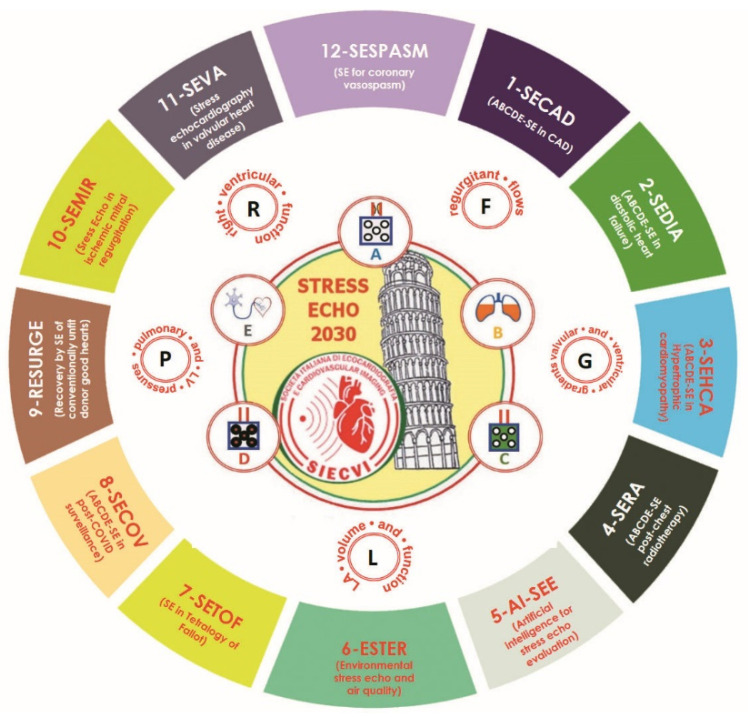
SE 2030 at a glance. The core protocol of SE2030 is the same as in SE2020 with ABCDE: epicardial coronary artery stenosis (with RWMA), step A; lung water (with B-lines), step B; myocardial function (with left ventricular end-systolic volume for contractile reserve and end-diastolic volume for preload reserve), step C; coronary microvascular dysfunction (with CFVR), step D; cardiac autonomic balance (with HRR), step E. Ancillary steps (necessary in some but not all patients) are step F (regurgitant flows), step L (left atrial volume and function), step P (pulmonary and LV pressures) and step R (right ventricular function). The study is endorsed by the Italian Society of Echocardiography and Cardiovascular Imaging and initiated in Pisa, Italy, as shown by the Leaning Tower present in the logo. SE protocols are indicated from 1 to 12 clockwise, and cover a wide spectrum of clinical conditions within and beyond CAD.

The various projects will include patients with known or suspected CAD (project 1), known or suspected heart failure with preserved ejection fraction (project 2), hypertrophic cardiomyopathy (HCM, project 3), status post-chest radiotherapy and chemotherapy (project 4), repaired Tetralogy of Fallot (project 7), cardio-pulmonary involvement post-COVID 19 (project 8), post-ischemic (project 10) and primary valvular heart disease (project 11), and suspected coronary vasospasm, a diagnosis frequently missed but important to recognize as a possible cause of life-threatening disease, which is easy to treat when promptly identified (project 12). The 12 protocols running on the SE-ABCDE platform are spread all over the spectrum of cardiovascular disease, from severe valvular heart disease to suspected CAD in patients with normal LV function. Potential heart donors with brain death will be evaluated to assess the suitability for donation of hearts currently dismissed on the basis of clinical history criteria but in the absence of a cardiac functional evaluation (project 9). The study will exploit and possibly contribute to upgrading the leading edge quantitative and operator-independent technology of AI-SE and cardiac strain (project 5) for image interpretation and data analysis and will also evaluate the results of SE parameters in the context of powerful environmental modulators of stress results and/or long-term outcome such as air pollutants and medical radiation exposure analyzed through big data mining with AI (project 6). The overarching aim of the study is to make SE practice more uniform, versatile, standardized, quantitative, and evidence-rich, producing data potentially relevant to change clinical practice (Figure 1).

When clinically indicated, SE will be repeated comparing results before and after treatment. Treatment will include medical treatment, percutaneous coronary interventions, transcatheter aortic valve implantation, surgical treatment, device therapy, and others.

### 2.5. Sponsored by a Professional Scientific Society

The study is investigator-driven and not industry-driven. It is endorsed by an independent-not for profit professional society (Italian Society of Echocardiography and Cardiovascular Imaging) and not sponsored by industry, although some materials useful for project completion such as AI-software will be donated by industrial partners for recruiting centers.

### 2.6. ABCDE-SE in CAD (SECAD)

#### 2.6.1. Background

The cornerstone of diagnosis with SE is the finding of reversible RWMA. This is the only sign established through over 40 years of clinical experience and endorsed in general cardiology guidelines [8]. However, the valuable diagnostic and prognostic information provided by SE extends well beyond RWMA [9] and today includes all the steps of the comprehensive ABCDE protocol, including B-lines [10], LVCR [11], CFVR [12], and HRR [13]. Functional mitral regurgitation (step F) is also important in patients with mitral regurgitation at least mild at rest since it may significantly worsen during stress affecting prognosis and possibly driving specific treatment [14,15,16], especially in patients with dilated cardiomyopathy. During inotropic stress (dobutamine and exercise, especially in upright position) step G will be assessed to identify dynamic left ventricular outflow tract obstruction as a cause of chest pain, dyspnea, or syncope [14,15,16].

#### 2.6.2. Aims

The primary aim is to evaluate the feasibility of rest and stress-induced integrated approach during SE with the ABCDE protocol with different stress modalities. The secondary aim is to assess the prognostic value of the different steps for predicting outcome.

#### 2.6.3. Methods

All patients (“all-comers”) referred to the SE lab with known or suspected CAD will be evaluated with ABCDE-SE. Presenting patients referred to SE lab according to existing 2020 guidelines indications and contraindications will be recruited (Table 1).

Patients with at least 14 readable segments on resting echocardiogram will be referred for: (a) assessment of chest pain or dyspnea; (b) risk stratification of (ischemic or non-ischemic) dilated cardiomyopathy; (c) for reassessment after an abnormal test or assessment of known CAD (previous acute coronary syndrome and/or previous myocardial revascularization, prior CAD by invasive or noninvasive coronary angiography); (d) for risk stratification prior to high or intermediate risk non-cardiac vascular surgery (such as liver transplant or major non-cardiac vascular surgery) in patients with poor functional capacity (<4 METS) and/or suspected cardiac symptoms, in presence of a revised cardiac risk index (Lee criteria) ≥2 (high risk surgery; CAD; congestive heart failure; cerebrovascular disease; diabetes mellitus on insulin; serum creatinine > 2 mg/mL); (e) status post heart transplant; (f) pediatric patients and congenital heart disease (Kawasaki, transposition of the great arteries/status post-arterial switch operation, anomalous origin of a coronary artery, familial homozygous hypercholesterolemia); (g) peri-partum cardiomyopathy. Usual contraindications will apply to all forms of stress testing as recommended by major scientific societies [7,9]: 1-unstable or complicated acute coronary syndromes; 2-severe cardiac arrhythmias (ventricular tachycardia, ventricular fibrillation, complete atrio-ventricular block). The presence of moderate to severe systemic hypertension (resting systolic blood pressure >180 mmHg) is a contraindication to exercise or dobutamine; the presence of a hemodynamically significant LV outflow tract obstruction (LV intraventricular gradient >30 mmHg) is a specific contraindication to dobutamine; significant hypotension (resting systolic blood pressure <90 mmHg) and pronounced active bronchospastic disease are a specific contraindication to vasodilator stress (dipyridamole or adenosine or regadenoson). Information on demographics (sex, age, body mass index, and body surface area), lifestyle (smoking and physical activity), other risk factors and ongoing therapy will be collected. From resting echocardiography and vascular carotid scan evaluation data related to carotid disease and cardiac calcification will also be collected, when available, since both may contribute significantly to atherosclerosis phenotyping and comprehensive risk stratification independently and incrementally to SE results [14]. All patients will enter a regular clinical follow-up program with annotation of cardiovascular and non-cardiovascular endpoints, since the biomarkers under investigation may precede and predict conditions other than cardiovascular disease, such as cancer or neurodegenerative disease, characterized by low grade inflammation, somatic DNA instability, endothelial dysfunction (step D positivity) and autonomic dysfunction (step E positivity) which are associated with SE positivity and are considered biomarkers of systemic, not merely cardiovascular, disease.

#### 2.6.4. Sample Size Calculation

The expected incidence of SE positivity (by at least one of the ABCDE criteria) is around 35% [10,11,12,13]. For prognostic end-point, we conservatively assume a 5% yearly incidence of composite end-points (all-cause death, myocardial infarction, stroke, progression of chronic heart failure which requires hospitalization, evident intensification of diuretic therapy, new hospital admission for heart failure, heart transplant, ventricular assist device implantation, aborted sudden death, or new onset atrial fibrillation or atrial flutter), with doubling of likelihood of events in the presence of a positive SE (for composite criteria). Assuming that the hypothesis of proportionality of hazard holds, as required for Cox proportional hazards regression, a sample size of about 2430 patients with a 5-year follow-up is required to provide 90% power with an alpha error of 5% to detect a difference for the primary endpoint of all-cause mortality among those with positive versus negative SE also considering a 20% drop-out. The estimated sample size will also allow detection of differences in secondary endpoints such as coronary revascularization for clinically refractory angina (predicted by A); readmission for decompensated heart failure or development of de novo heart failure (step B and step C); development of heart failure (step D); sudden death, de novo atrial fibrillation, severe ventricular arrhythmias such as sustained ventricular tachycardia causing syncope or ventricular fibrillation (step E). The newly recruited patients will add-up to the patients already recruited in years 2016–2020 with the same methodology as a part of a similar project (named DITSE) in SE2020 (1).

#### 2.6.5. Study Hypothesis

ABCDE-SE is highly feasible with excellent success rate in all patients with all stress modalities.

The five steps of ABCDE protocol have independent and incremental value in predicting outcome, and each one selectively predicts some endpoints providing an integrated assessment of the many possible vulnerabilities of the patient. Each phenotype (ischemic, congestive, failing, microcirculatory, autonomic) is especially responsive to specific therapies which are left to the decision of referring physician and will be recorded in the follow-up to allow exploratory analysis.

### 2.7. ABCDE-Stress Echo in Diastolic Heart Failure (SEDIA Project)

#### 2.7.1. Background

Diastolic heart failure is also called heart failure with preserved ejection fraction and is a challenging target of diastolic SE [15,16]. The diagnosis remains difficult, and the previous European Society of Cardiology criteria, based upon low sensitivity criteria such as echocardiographic data and plasma natriuretic peptides [17], have been recently revised with a new stepwise approach and a pretest assessment (including resting transthoracic echocardiography) in any patient with symptoms or signs compatible with heart failure, progressing to diastolic SE for intermediate score values and finally moving to invasive rest and exercise hemodynamic study for final confirmation of diagnosis. A pulmonary capillary wedge pressure ≥15 mm Hg at rest or ≥25 mm Hg at peak exercise is diagnostic of heart failure with preserved ejection fraction [18].

This stepwise approach has some potential limitations in the reliance on costly, risky, and time-consuming techniques such as rest and exercise invasive hemodynamic testing. They were not practiced in any of the 30 laboratories of SE2020 network as a further demonstration of the dissociation between the aerial world of guidelines and ground-based clinical practice populated by restrictions due to economic, logistic, and medico-legal concerns. A much simpler clinical score can be used with parameters such as age, obesity, atrial fibrillation, anti-hypertensive treatment, and resting echocardiographic parameters such as echocardiographic E/e’ ratio >9 and systolic pulmonary artery pressure >35 mm Hg, which however misses the heterogeneity of clinical and echocardiographic responses during stress of patients with identical clinical presentation and resting echocardiographic variables [19]. Indeed, more than one-half of patients with heart failure with preserved ejection fraction have normal/near normal left ventricular filling pressures at rest and invariably require stress to bring out the heart failure phenotype [20]. The 2019 European Society of Cardiology algorithm does not provide any indication for patients unable to exercise, and yet exercise is not feasible in about 50% of patients with the epidemiological profile of heart failure with preserved ejection fraction (age > 70 years, obesity/overweight, hypertensive, and diabetic). The algorithm does not include the recently developed parameters which focus on key aspects of diastolic heart failure physiology such as reduced preload reserve [21], coronary microvascular disease as a trigger and amplifier of myocardial fibrosis in the natural history of the disease [22], and blunted chronotropic reserve with Step E of stress testing [23]. It emphasizes the value of indices such as E/e’ and tricuspid regurgitant jet velocity during exercise with known limitations of feasibility (50%) and accuracy, with unsatisfactory correlations with invasively measured parameters that they are supposed to mirror such as LV end-diastolic pressure for E/e’ and pulmonary artery systolic pressure for tricuspid regurgitant jet velocity [24].

Patients with unexplained dyspnea often have occult cardiac causes which can be easily unmasked by SE such as inducible ischemia, severe mitral regurgitation, or dynamic LV outflow tract obstruction [16]. These patients should be removed from the basket of heart failure with preserved ejection fraction since they exemplify a different pathogenesis and require a different therapy [16]. While E/e’ and tricuspid regurgitant jet velocity may play a role in characterizing changes in hemodynamics during stress, SE has much more to offer in the screening, diagnosis, risk stratification, and therapy in patients with known or suspected heart failure with preserved ejection fraction. Emerging data suggest left atrial mechanics may play a significant role in proportion of patients with heart failure with preserved ejection fraction, particularly in those with atrial dysrhythmias. While left atrial volume index at rest may convey risk of heart failure with preserved ejection fraction, changes in left atrial volume with stress may better risk stratify patients with 1 out of 5 patients seeing a reduction in left atrial volume with stress, but another 1 out of 5 with normal left atrial size dilating during stress [25]. Left atrial strain, particularly reservoir strain is also feasible, reproducible, and important to characterize left atrial function during stress. Pulmonary artery systolic pressure can be measured in virtually all patients during exercise if we use acceleration time of pulmonary flow velocity for patients with unreadable tricuspid regurgitant jet velocity [26]. A steep increase of left ventricular filling pressure and pulmonary capillary wedge pressure during exercise is a typical hemodynamic response in heart failure with preserved ejection fraction [27] This elevated pressure then backwardly transmitted to pulmonary circulation results in pulmonary congestion. This, in turn, overloads the right ventricle as well, causing right ventricular dysfunction and failure. In heart failure patients right ventricular free wall strain performs better (in terms of diagnostic and prognostic power) than conventional echocardiographic measures in the detection of right ventricular dysfunction [28]. Volumetric SE allows to measure preload reserve as the increase in end-diastolic volume and the contractile reserve through the reduction in end-systolic volume. Rest and stress B-lines are essential to recognize a pulmonary congestion phenotype [29] which may require B-lines driven decongestion therapy [30]. Therefore, ABCDE-SE during exercise or pharmacological SE deserves a systematic assessment in this challenging cohort with three major objectives: 1-to screen and exclude cardiac causes of dyspnea mimicking diastolic dysfunction; 2-to identify the reduction of functional reserve in cardiac output and its underlying separate but not mutually exclusive mechanisms (reduction in chronotropic, preload, or contractile reserve); 3-to characterize the underlying heterogeneous phenotypes potentially allowing a targeted therapy, from pulmonary congestion to coronary microvascular dysfunction.

#### 2.7.2. Aims

The primary aim is to evaluate the feasibility and value of a SE-centered approach to diagnose heart failure with preserved ejection fraction in patients who can exercise, after screening for common mimickers of this condition such as severe mitral regurgitation, inducible ischemia, and dynamic LV outflow tract obstruction. The secondary aim is to evaluate the feasibility of a SE-centered approach with pharmacological stress in patients who are unable to exercise, which represent a high proportion of the total population of heart failure with preserved ejection fraction patients. The tertiary aim is to assess the prognostic value of SE indices (ABCDE plus pulmonary artery systolic pressure with tricuspid regurgitant jet velocity or acceleration time of pulmonary flow velocity, right ventricular free wall strain, left atrial volume and strain) for outcome stratification, compared to standard predictors such as plasma cardiac natriuretic peptides levels.

#### 2.7.3. Methods

Patients with dyspnea and known or suspected heart failure with preserved ejection fraction by 2019 European Society of Cardiology criteria will be enrolled and studied with cycle-ergometer in semi-supine SE (or treadmill). A score of at least 1 according to the criteria proposed by Pieske et al. is required for inclusion [18]. The score (from 0 to 9) proposed by Reddy et al. on the basis of simple clinical and resting echocardiographic parameters will be also assessed [19]. In patients unable to exercise or when exercise is not feasible or did not allow sampling of CFVR, pharmacological test (vasodilator or dobutamine) is recommended. The diastolic assessment should be included into all exercise SE tests by measuring standard Doppler-derived mitral inflow velocity, pulsed Tissue Doppler of mitral annulus, and retrograde tricuspid gradient of tricuspid regurgitation. These measurements can be performed at intermediate load of exercise and/or 1–2 min after the end of the exercise, after obtaining wall motion acquisitions, when the heart rate decreases and mitral inflow E and A velocities appear to be well separated. As a part of the “diastolic package”, we will also assess, at baseline, intermediate load (50 watts) and peak-post stress (11): end-diastolic left ventricular volume index (to evaluate left ventricular diastolic volume reserve, impaired in stiff hearts, which are less dilated for any given filling pressure); end-systolic left ventricular volume index (for assessment of left ventricular force, which may unmask occult systolic dysfunction with normal ejection fraction increase); ejection fraction and both stroke volume and cardiac output (to assess conventional contractile reserve) from 2D images; mitral regurgitation and left ventricular outflow tract obstruction; pulmonary artery systolic pressure (from velocity of tricuspid regurgitation or acceleration time of antegrade systolic pulmonary flow); B-lines with stress (to provide a direct imaging of extra-vascular lung water accumulation as a direct cause of dyspnea); right ventricular free wall strain to assess the presence of right ventricular dysfunction; left atrial volume index (with an abnormal response identified as a dilated left atrium exceeding the physiologic preload reserve or a stiff heart failing to dilate with increased pressures witnessed by B-lines increase); peak atrial longitudinal strain (a highly feasible and reproducible index of atrial reservoir function); and mitral inflow E velocity and mitral annulus e’ tissue Doppler velocity. Global longitudinal strain (GLS) will be determined as the average of the regional longitudinal strain measured in a 16 or 17 segment model from the apical long-axis, 4-chamber, and 2-chamber view.

#### 2.7.4. Sample Size Calculation

The expected incidence of composite end-points (as defined in project 1) is around 20% per year. We assume a positivity rate to SE (by composite criteria) of 30%, with doubling of likelihood of events in presence of SE positivity (by any criteria). Assuming that the hypothesis of proportionality of hazard holds, as required for Cox proportional hazards regression, with a power of 90%, an attrition rate of 10% and a 5-year follow-up period of a sample size of 181 patients is required. The newly recruited patients will add-up to the patients already recruited in years 2018–2020 with the same methodology as a part of the same project in SE2020 [1].

#### 2.7.5. Study Hypotheses

The invasive hemodynamic-based diagnosis of heart failure with preserved ejection fraction is not feasible for routine practice. The current clinical diagnostic criteria are of variable quality and not well tested in large patient populations. A comprehensive non-invasive stress-based algorithm have should be feasible, safe, simple, and prognostically relevant, thereby leading to “replace, reduce, and refine” (the 3 R’s approach) the current criteria: Replace invasive with noninvasive, ionizing with nonionizing, and rest with stress evaluation; Reduce the number of patients labelled heart failure with preserved ejection fraction by identifying at the outset patients with inducible ischemia presenting as dyspnea as the main symptom or with stress-induced mitral regurgitation or left ventricular outflow tract obstruction; Refine the current sub-setting identifying different phenotypes on the basis primarily of cardiac functional reserve (possibly impaired for chronotropic, preload, or contractile reserve) and associated phenotypes such as pulmonary congestion or coronary microvascular disease.

### 2.8. Stress Echo in Hypertrophic Cardiomyopathy (SEHCA)

#### 2.8.1. Background

HCM is a heterogeneous inherited cardiomyopathy with variable phenotypic expression [31,32,33]. The assessment of mortality risk in asymptomatic or mildly symptomatic patients is a challenging task, and several approaches targeted on different physiologic variables have been proposed. Resting transthoracic and SE are especially attractive for the purpose of identification of different phenotypes in HCM, risk stratification and serial follow-up examinations are often needed in the same patient to assess natural history and effects of interventions [15,16]. As a consequence, facilities and skills for exercise SE are usually available in specialist HCM centers [32,33]. Exercise SE provides comprehensive information on the different vulnerabilities of the HCM patient with the ABCDE protocol: RWMA due to myocardial ischemia [34], pulmonary congestion due to diastolic dysfunction, preload reserve and contractile reserve impairment, coronary microcirculatory dysfunction [35], and blunted HRR which is a marker of cardiac autonomic dysfunction and reduced sympathetic reserve [36]. In addition to this standard ABCDE protocol adapted to HCM, at least two other parameters can be added in the assessment of HCM: evaluation of regurgitant mitral flow (step F) and dynamic left ventricular outflow tract gradient (step G) [37]. Of note, exercise limitation and breathlessness may be due to a number of different causes. Despite similar clinical manifestations, management may differ substantially based on the mechanisms [38]. SE is the only test with the potential to discriminate the various components, allowing a targeted treatment driven by pathophysiology.

#### 2.8.2. Aims

The primary aim is to evaluate the feasibility of comprehensive ABCDEFG-SE in the evaluation of HCM. The secondary aim is to assess the value of each of these parameters in predicting response to specific therapy and other interventions. The tertiary aim is to assess the prognostic value of SE indices for prognostic stratification in the medium-long-term.

#### 2.8.3. Methods

Diagnosis of HCM will be based on existing guidelines [31]. Phenocopies such as infiltrative/storage disease (e.g., Fabry, amyloid) will be excluded. All patients will be followed-up and the prognostic value of different rest and SE parameters (also compared to standard prognostic indices) will be assessed. For each patient new or changing therapies will be recorded and symptomatic status reassessed every year as unchanged (same New York Heart Association class), improved (class decrease ≤ 1), or worsened (class increase ≥ 1). In patients and first-degree relatives with genetic characterization already performed as a part of the routine work-up different phenotypes will be correlated with specific genotypes. Non-imaging or routine imaging non-ultrasound exams such as EKG, cardiovascular magnetic resonance (myocardial fibrosis with delayed enhancement), and other available examinations will be collected and analyzed also with neural network analysis techniques developed in project 5.

#### 2.8.4. Sample Size Calculation

The composite outcome end-points of the study are defined as in project 1 plus myectomy and percutaneous transluminal septal myocardial ablation. A pilot study showed an incidence of events around 8% per year [37]. Hypothesizing assumptions behind the Cox proportional hazards regression are met, if we assume a positivity rate to SE (by composite criteria) of 40%, a 5-year follow-up doubling of likelihood of events in presence of SE positivity (by any criteria), with a power of 90%, an alpha error of 5%, and an attrition rate of 10%, a sample size of 338 patients is required. The newly recruited patients will add-up to the patients already recruited in years 2018–2020 with the same methodology as a part of the same project in SE2020 [1].

#### 2.8.5. Study Hypothesis

HCM patients have different phenotypes with a spectrum of different underlying functional alterations and therapeutic correlates. SE-ABCDEFG is essential to identify the pathophysiologic and prognostic heterogeneity underlying similar clinical manifestations allowing targeted therapeutic actions.

### 2.9. Stress Echo Post-Radiotherapy (SERA)

#### 2.9.1. Background

Radiation-induced heart disease is associated with a significantly higher morbidity and mortality in cancer patients [39]. It affects cancer survivors who received chest radiation therapy as an adjuvant or exclusive treatment for cancer. The most frequent forms treated with chest radiation therapy are breast, lung, and esophageal cancers or lymphoma. Less frequently, pleural mesothelioma and thymic malignancies are treated with chest radiotherapy. Radiotherapy is based on photon therapy (with conventional or advanced protocols) or proton therapy [40]. The chances of developing radiation-induced heart disease increase with higher cumulative doses (>30 Gray) in anterior or left sided irradiation, concomitant chemotherapy (especially cardiotoxic anthracycline therapy), presence of cardiovascular risk factors, and with increased distance from time of irradiation. SE is recommended in these patients for several reasons. First, alternative excellent imaging tests are available but they require exposure to ionizing radiation or potentially toxic agents. It is especially important to use safe and nonionizing diagnostic modalities in these cancer patients who need serial follow-up examinations. These patients already developed a primary cancer, and they previously received radiotherapy which predisposes to second cancer. In all patients, and in these patients in particular, every dose counts to determine cumulative exposures and therefore extra-risk of cancer. Second, the presentation is often vague, years or even decades after chest radiation exposure, and requires a high index of suspicion with comprehensive assessment to allow early detection which may allow timely treatment often with percutaneous interventions. Third, there is not a single target of radiation-induced heart disease but many different pathophysiological targets which can be recognized with a comprehensive SE approach. Radiation-induced coronary atherosclerosis is the major clinical effect in post-radiotherapy patients. The estimated incidence of major cardiac events related to ischemic heart disease is 30% at 10 years post-treatment in female patients with radiotherapy post-breast cancer [41]. Radiation-induced inflammatory response and direct DNA damage are associated with endothelial dysfunction and smooth muscle cell proliferation leading to macrovascular damage and accelerated atherosclerosis with inflammatory plaques with high collagen and fibrin content. The resulting epicardial artery stenosis may be identified also at a pre-symptomatic or asymptomatic stage as possible abnormalities of step A of SE. Low grade inflammation determines increased permeability of the alveolar capillary barrier favoring lung congestion and abnormalities of step B, also possible for rarefaction and fibrosis of lung lymphatic vessels and lung fibrosis [42]. Progressive myocardial fibrosis may lead to systolic and/or diastolic dysfunction altering step C [43,44], which is also impaired in case of pericardial constriction which selectively alters preload reserve. Microvascular injury and reduced myocardial capillary density decrease coronary microvascular vasodilatory capacity [45] and therefore may alter step D. Neuronal cell inflammation and degeneration of extrinsic and intrinsic (intrapericardial) autonomic nervous system can determine alterations in autonomic balance with inappropriate sinus tachycardia or reduced sympathetic reserve detectable with step E [46]. The standard ABCDE protocol is also further expanded in these patients to F, G, P, and R steps to face the complexity of multifaceted damage. Valve leaflets, fibrosis, and accelerated calcification can lead to significant, mostly mitral, regurgitant flows (step F) and mostly aortic stenosis with transvalvular gradients (step G) [47]. Pulmonary circulation can show a significant rarefaction and increased reactivity assessed with pulmonary vascular resistances (step P) and right ventricle (step R) can show significant alterations in structure and function [48]. The same clinical manifestation of dyspnea can recognize extremely heterogeneous conditions which may limit quality of life and survival in these patients, and therefore a comprehensive assessment is needed for a targeted therapy.

The application of SE in post-radiotherapy patients is recommended by scientific societies [49,50,51]. For asymptomatic patients with a history of mediastinal chest radiation, major imaging societies recommend a screening transthoracic echocardiography and SE at 10 years after mediastinal radiation therapy and serial exams every 5 years thereafter. The National Comprehensive cancer network has similar period recommendations for SE [50]. As stated by European Society of Medical Oncology recommendations 2020, “nonionizing modalities may be most appropriate due to concern regarding cumulative radiation dose in cancer patients”, who are already highly exposed for oncology diagnosis and follow-up programs [51]. Additionally, SE has a role in the assessment of potentially cardiotoxic chemotherapies. It can be helpful for the detection of subclinical LV dysfunction, in addition to allowing detection of accelerated atherosclerosis.

#### 2.9.2. Aims

The primary aim is to assess the feasibility of an integrated ABCDEFG (+PR) approach in these patients. The secondary aim is to evaluate other SE parameters in populations stratified according to radiotherapy (type, location, cumulative dose, combined chemotherapy), chemotherapy, and clinical variables (age at exposure, cardiovascular risk factors, genetic substrate when available). The tertiary aim is to assess the prognostic value of individually considered or combined SE indices in prognostic modeling using traditional risk factors and radiotherapy variables.

#### 2.9.3. Methods

ABCDEFG (+PR) SE will be performed and analyzed according to the general standardized protocol. Inclusion criteria: 1-history of photon or proton radiotherapy 10 years or more in asymptomatic patients; 2-history of chest radiation therapy in symptomatic patients (dyspnea, chest pain, palpitations); 3-history of chest radiation therapy in asymptomatic patients with significant alterations (>mild) in resting TTE (such as ejection fraction <40%, diastolic dysfunction, constrictive physiology). The relevant radiotherapy parameters will be collected and analyzed under the coordination of a radiation oncologist. For each patient, the minimum data set will include 8 factors generating a composite radiotherapy score (each item absent, score 0, to present, score 1, with overall values from 0, least cardiac vulnerability, to 8, most cardiac vulnerability). The 8 items are [45]: 1-younger age at exposure (<40 years, score 1); 2-overall dose (>30 Gray, score 1); 3-division into fractions >2 Gray (present, score 1); 4-the heart was exposed to ionizing radiation (yes, score 1); 5-Use of cytotoxic therapies (yes, score 1); 6-Irradiation technique (tele-radiotherapy, score 1, since it is more toxic than brachytherapy or proton therapy); 7-Radiation in the morning hours between 6 a.m. and noon (yes, score 1); 8-Longer time since exposure (>10 years, score 1).

#### 2.9.4. Sample Size Calculation

The composite outcome end-points of the study are defined as in project 1. A pilot study showed an incidence of events around 8% per year [6]. If we assume a positivity rate to SE (by composite criteria) of 20%, with doubling of likelihood of events in presence of SE positivity (by any criteria), with a power of 90%, an alpha error of 5%, and an attrition rate of 10%, a sample size of 507 patients is required.

#### 2.9.5. Study Hypothesis

Post-radiotherapy patients have different phenotypes with a spectrum of different underlying functional alterations and therapeutic correlates. SE-ABCDEFG is essential to identify the pathophysiologic and prognostic heterogeneity underlying similar clinical manifestations allowing targeted therapeutic actions.

### 2.10. Artificial Intelligence Stress Echo: AI-SEE

#### 2.10.1. Background

AI in echocardiography describes the applications of the overlapping fields of machine learning, deep learning, and network analysis to the processing and analysis of cardiac ultrasound images [52]. This field holds great potential in the development of self-learning algorithms capable of stratifying disease by providing clinicians with real-time analysis of medical images. AI may provide a solution for automated and in-depth handling of imaging and non-imaging information, with two main aims: 1-to make objective what is currently done by the ‘naked eye’, for instance, regional wall motion analysis, or by ‘hand measurements’, for instance, ejection fraction from left ventricular volumes (subproject: AI-SEE images); 2-to uncover complex clinical relationships that redefine disease and previously unseen, are undetectable by “natural” intelligence and conventional analysis models, can be extracted from data set through data mining and can be made readily available for clinical use (subproject: AI-SEE data) [53].

#### 2.10.2. AI-SEE Images

At present, operator-dependence remains a leading limitation in the interpretation and analysis of SE. An expert reader is substantially more accurate than a beginner reader [54]. While substantial training and a high volume of SE is required to reach [55,56] and maintain competence [57,58], low accuracy has been reported in high volume centers [59]. At present, current training and competency requirements are focused on the challenging analysis of RWMA, with contemporary practice requiring additional expertise, such as lung sonography. AI algorithms provide a platform with a high capacity for the automated analysis of complex and multifactorial data, which can identify pertinent and prognostic information relevant for the clinician. As such, AI has the capacity to mine image and non-image data to identify inter-related variables, which may optimize risk stratification for individual patients [60]. AI in SE (AI-SEE) may rapidly change the daily practice of echocardiography laboratories and likely the practice of cardiology, allowing integration, quantification, and operator-independence, so that echocardiography and SE can be established as the definitive, quantitative, unsupervised, and objective imaging test [61]. For individual centers, enhanced precision and reproducibility derived from AI means volumes of activity will no longer be necessary to guarantee the quality of the laboratory. For the individual reader, echocardiographic analysis time will be shifted from tedious measurements and time-consuming training to integration and innovation. As such, research into the value of AI for clinicians, echocardiographers, and importantly, the impact on patient care and outcomes in the real world is required. The SE2020 and SE2030 effectiveness study is a valuable platform to perform this work.

#### 2.10.3. AI-SEE Data

The overwhelming depth of information that can now be extracted from SE can be confusing for the cardiologist. In a 15 to 30-min examination we obtain unique data on cardiovascular anatomy, function, flow, structure, coronary supply, and lung appearances, with different techniques including M-mode, 2D, color-, continuous wave-, pulsed, tissue-Doppler, contrast, 3D, and deformation (strain) imaging. AI may help to mine big data, extracting information now hidden under the overflow of data with techniques such as machine learning and network analysis to identify inter-related variables, and thereby optimize risk stratification for individual patients.

#### 2.10.4. Aims

The two methodologically and conceptually interconnected projects have two separate aims:

AI-SEE images. Evaluate the prediction of patient outcomes from fully automated echocardiographic imaging algorithms in a large multicenter population undergoing SE.

AI-SEE data. To identify the hidden links between clinical imaging and stress variables and develop a tailored personalized model for risk prediction with specific biomarkers linked to specific endpoints.

#### 2.10.5. Methods

AI-SEE images. A total of 5000 consecutive studies will be enrolled from 20 centers over 3 years (250 from each center), between 2017–2023. All images (rest and peak stress) will be acquired in DICOM format and sent to core laboratory for analysis. Recruiting centers will have access to CE-marked AI driven software, providing clinicians with on-line risk stratification for CAD using algorithms in a cloud-based system. Data from SE2020 and SE2030 will be used to develop and refine existing AI algorithms for a two part statistical analysis; (1) the incremental value of machine learning algorithms to predict patient outcomes in comparison to routine clinical and SE data; (2) the incremental value of machine learning algorithms to predict patient outcomes with the 5-step ABCDE pathway [6] (A—regional wall motion; B—lung sonography, C—contractile reserve; D—rest and stress pulsed-wave Doppler tracings [with at least 3 beats]; and E—rest and stress echo EKG), with data provided by centers. For each parameter assessment (positivity versus negativity), the area under the receiver-operating characteristic curve produced by the automated parameters will be compared to that produced by the experienced cardiologist (cross-sectional analysis). Core lab will analyze data obtained with the ABCDE protocol in 5000 patients provided from the SE2020 (n = 2500, years 2017–2020) and SE2030 (n = 2500, years 2021–2023) projects in patients with known or suspected CAD. Data analysis will be performed with standard Cox multivariate analysis to assess the independent and incremental value of any AI variables compared to clinical and routine echocardiographic parameters as per the two-part statistical analysis. The input function will be the excel file with 5000 patients with follow-up information of at least 1 year. The analysis will evaluate independent predictors of all-cause mortality as the primary endpoint.

#### 2.10.6. Sample Size Calculation

AI-SEE images. Using the C-index of the Cox analysis, a sample of 626 will be sufficient to detect an incremental change in model performance of 0.05. Furthermore, assuming 5% mortality, the proposed sample size will be sufficient to avoid overfitting of the regression coefficients in the proposed analysis.

AI-SEE data. From previous similar experiences [62], a set of data (with ABCDE information) from 2500 patients acquired from at least 10 laboratories will be sufficient to develop the algorithm (modeling set) subsequently prospectively tested on a different set of 2500 patients (validation set).

#### 2.10.7. Study Hypotheses

AI-SEE images. AI allows an operator-independent assessment of RWMA and left ventricular volumetric data without loss of information compared to experienced cardiologist. AI-SEE data AI provides independent and incremental value to the ABCDE protocol for the prediction of all-cause mortality in patients undergoing SE for the assessment of ischemic heart disease.

### 2.11. ESTER: Environmental Stress Echocardiography, Air Pollution and Medical Radiation

#### 2.11.1. Background

Air pollution contributes substantially to cardiovascular morbidity and mortality [63]. Worsening of air quality induced by pollution acutely (same-day) increases the admission rates for acute coronary syndromes, acute decompensated heart failure, and atrial fibrillation [64,65]. Conversely, improvements of air quality reduce the admission rates for the same conditions, as has been proven during the COVID-19 pandemic [66,67] due to the plummet of air pollution because of lockdowns [68].

Changes in hospital admissions are only the tip of the iceberg of cardiovascular toxic effects of pollution, since in chronic conditions patients with CAD or heart failure may show an increased vulnerability to inducible ischemia. In particular, fine particulate matter and nitrogen dioxide concentration may affect cardiovascular function through the increase in inflammatory and oxyradical stress possibly favoring the induction of myocardial ischemia [69], diastolic dysfunction and pulmonary congestion, functional LV abnormalities leading to reduced contractile reserve, coronary microvascular inflammation, and cardiac autonomic unbalance [70] with blunted sympathetic reserve [71,72]. Each functional abnormality is a possible substrate of prognostic vulnerability, and all of them can be simultaneously evaluated under controlled conditions with ABCDE protocol by exercise or pharmacological SE. This is especially important since air pollution is also an actionable therapeutic target. Fine particulate matter can be decreased by 50% with air purifiers, with a 68% reduction in inflammatory markers such as interleukin-1 and significant 3 to 5% reductions in systolic and diastolic blood pressure [73]. Face mask respirators approved by the National Institute for Occupational Safety and Health fit tightly to the face and filter at least 95% of airborne particles, including aerosolized nano-particulates [74]. Any condition which reduces the individual exposure to toxic air pollutants such as fine particulate matter or nitrogen dioxide can therefore reduce the vulnerability of the patient to the various manifestations of environmental changes as a cardiovascular stress [75]. It is not clear at present which pollutant or mixture of pollutants is more toxic to the heart (with nitrogen dioxide and fine particulate matter on the top of the list); which cardiovascular function is most affected in which patients (although alveolar capillary membrane and coronary microcirculation seem to be the privileged target in heart failure and CAD patients); and if the changes in air quality (achievable with outdoor or indoor or individual protection devices) can affect the vulnerability of myocardium in the short term, under controlled conditions, providing the cardiologist with an actionable low cost and potentially highly effective therapeutic tool, allowing to clean the air to treat the heart. In addition to air pollution data, also data on personal medical radiation history will be collected, since ionizing radiation is a recognized risk factor for cancer and atherosclerosis and cumulative doses reach significant values in cardiological patients with a risk increase of the same stochastic type and similar mechanisms of epigenetic DNA damage and low-grade inflammation as air pollution [76,77].

#### 2.11.2. Aims

The primary aim is to assess the inter-patient correlation between SE results and outdoor air pollution levels in patients matched for clinical, coronary anatomy (if available) and resting functional features. The secondary aim is to assess the effects of air quality and cumulative medical radiation exposure in prognostic modeling using traditional risk factors and SE results.

#### 2.11.3. Methods

All patients enrolled in projects 1 to 4 (CAD, heart failure with preserved ejection fraction, HCM, post-radiotherapy) undergoing clinically-driven ABCDE-SE also have information on house residency and work place in the data bank. On the basis of this information, the air epidemiology unit will obtain same day local air quality data from publicly available data sets from the regional authority of environmental protection. Due to disparate regulatory conditions in the countries participating in the study, reliable and consistent access to publicly available data sets is expected in 50% of recruited patients. For each patient and each test of the same patient, the values of 2 particulate and 4 gaseous pollutants will be collected when available: fine particulate matter with aerodynamic diameter <2.5 µm (fine particulate matter), particulate matter with aerodynamic diameter <10 µm, nitrogen dioxide, ozone, carbon monoxide, and sulfur dioxide. Values of the same day, geo-referenced on the SE laboratory location and averaged values of the previous 30 days geo-referenced on the patient home will be taken as representative of that specific condition using the air monitor point closest to the echocardiography laboratory (for same day sampling) or patient’s home (for 30-days sampling). If the test is carried out in the morning, the average of the concentrations recorded between 8 a.m. and 1 p.m. will be used. If the test is performed in the afternoon, the concentrations between 2 p.m. and 7 p.m. will be considered. If hourly data are not available, daily averages will be used. When the values are not available for malfunction or other reasons, the previous day or following day values will be considered. Air quality data will be collected and inputted by an assessor unaware of the patient identity, condition, and functional test findings. Values of humidity (%), temperature (degrees C) and atmospheric pressure will also be collected when available.

At the moment of testing, data on medical radiation exposure will also be systematically collected. The cumulative radiation exposure reaches significant values in adult cardiology patients [78] and is an environmental risk factor for subsequent development of cancer and CAD, with a potential synergic effect with air pollution and other risk factors. Cancer risk is stochastic (the frequency of the event depends on the dose) for any dose (without threshold) and cardiovascular effect is considered deterministic (tissue reactions) for organ doses above the threshold of 500 milligrays according to the recent recommendations of the International Commission on Radiological Protection [79]. SE positivity for wall motion [80] and CFVR criteria [81] is a risk factor for subsequent development of cancer but it remains unclear if this association is mediated by common biological roots of epigenetic DNA damage between CAD and cancer or the iatrogenic effect of substantial diagnostic and therapeutic radiation exposure in advanced CAD and heart failure. The exposure will be quantified with the number of high radiation exposures collected by the patient prior to testing. Higher dose exposures (effective dose >5 millisievert corresponding to the equivalent of 250 chest X-rays) are invasive (such as coronary angiography) and noninvasive (such as radionuclide myocardial perfusion scintigraphy) and will be collected in radiological history to build a simple radiation exposure score with the number of lifetime diagnostic and therapeutic exposures as a single index. The number of procedures with significant radiation exposures is a proxy of cumulative medical radiation exposure and is a risk factor for cardiovascular disease and cancer both in pediatric [82] and adult heart patients [83]. Information regarding ionizing radiation procedures and—when available—patient dose values is acquired from either retrieval for hospital PACS system or from paper medical records accessed by the physician. The radiological risk score will be obtained combining the number of procedures and the dose of each procedure, derived from reference doses or-when available—from direct reading of the dose from the PACS system converted into effective dose with appropriate conversion factors. For instance, for cardiac catheterization a factor of 0.21 may be used to convert the Gray.cm^2^ into millisievert (effective dose) [76,77].

#### 2.11.4. Sample Size Calculation

Of the 2 430 tests recruited in protocol 1, at least 600 will have access to geo-referenced air quality data. This will allow to assess the value of individual air parameters on individual SE parameters. In particular, on the basis of a pilot study a 25% increase in air nitrogen dioxide concentration is expected to induce a 10% increase in abnormal CFVR results. This difference will be detected with a power of 90% and an alpha error of 5%, a sample size of 523 patients for the inter-patient assessment.

### 2.12. SETOF Stress Echo in Operated Tetralogy of Fallot

#### 2.12.1. Background

Pediatric SE is increasingly used in children and adults for the detection of myocardial ischemia (see project 1) and for functional characterization and risk stratification of grown-up patients with congenital heart disease [84]. The lack of radiation exposure is especially important in these patients who already receive an intensive radiation exposure with associated increased risk of cancer [85]. Tetralogy of Fallot is the most common cyanotic congenital heart lesion, and since treatments became available over 70 years ago, there are now a large number of patients with repaired Tetralogy of Fallot. After Tetralogy of Fallot repair, children often have residual lesions (the most common being pulmonary regurgitation) which can be treated with surgical or catheter-based pulmonary valve replacement decreasing right ventricular size, but this is not yet correlated with improved outcome. Pulmonary regurgitation can cause progressive right ventricular dilatation and dysfunction. In patients with Tetralogy of Fallot, morbidity and mortality are strongly related to right ventricular dysfunction. For this reason, the early detection of right ventricular dysfunction before it reaches an irreversible stage remains crucial. Unfortunately, resting parameters have shown a limited ability to detect early impairment of right ventricular function. Recently, a few studies have suggested that physical or pharmacological stress may unmask abnormalities of right ventricular function in patients with repaired Tetralogy of Fallot, with normal right ventricular function under resting conditions [86,87]. SE allows the simultaneous assessment of right and left ventricular global and regional function and Doppler parameters as well as coronary flow reserve in posterior descending coronary artery and chronotropic reserve [88] can be impaired in these patients and are potential determinant of exercise capacity [89].

#### 2.12.2. Aims

The primary aim is to evaluate the feasibility of right ventricular SE in patients with repaired Tetralogy of Fallot. The secondary aim is to assess the presence and amount of right ventricular contractile reserve and its correlation with indices of functional severity (NYHA class, cardiac natriuretic peptides, peak VO2, 6-min walking test, etc.). The tertiary aim is to assess the prognostic value of SE indices for prognostic stratification in the medium and long-term.

#### 2.12.3. Methods

Patients with repaired Tetralogy of Fallot or Fallot-like pathology (double-outlet right ventricle Fallot type, Tetralogy of Fallot with pulmonary atresia), evaluated at least 1 year after the last surgical or percutaneous procedure, will be recruited by regional reference centers for congenital heart disease. Additional inclusion criteria are age > 10 years, height > 140 cm, New York Heart Association class I or II. Right ventricular function will be assessed at baseline and peak stress with variations (rest and peak stress) of tricuspid annular plane systolic excursion, an index of right ventricular longitudinal function, and right ventricular fractional area change (a load-dependent index of right ventricular inlet function). Due to the influence of load on these measures, they tend to reflect right ventricular arterial coupling rather than measures of right ventricular contractility per se. To distinguish between genuine right ventricular dysfunction and/or pathological increases in pulmonary vascular load, whenever possible we will combine systolic pulmonary artery pressure and right ventricular end-systolic area using echocardiography to calculate right ventricular end-systolic pressure-area relation as a surrogate of right ventricular contractility. Peak systolic tricuspid annulus velocity and conventional indices of left ventricular systolic and diastolic function will also be measured at baseline and peak stress according to the standard ABCDE-FGLPR protocol. Right ventricular free wall strain alone and combined with interventricular septum strain will be assessed since these advanced imaging parameters have proved especially useful in predicting a reduced exercise tolerance in these patients even when performed at rest [90]. Resting right atrial strain will also be included in this analysis. Left ventricular function will also be assessed through measurement of ejection fraction, wall motion score index and E/e’ at baseline and peak stress. At the moment of testing, also data on medical radiation exposure will be systematically collected with the generation of a simple radiological risk score as detailed in project 6, with specific values of effective dose available for pediatric exposures associated with higher biological effects for any given physical dose exposure [85].

#### 2.12.4. Sample Size Calculation

The expected incidence of SE positivity (by increase in tricuspid annular plane systolic excursion <5 mm) is around 30% as shown by previous pilot studies [7]. With a power of 90%, an alpha error of 5%, and an attrition rate of 10%, a sample size of about 250 patients is required to detect a significant stress-induced increase in tricuspid annular plane systolic excursion. For the exploratory prognostic analysis (tertiary end-point), we conservatively assume a 20% yearly incidence of the predetermined end-points defined as in project 1, with doubling of likelihood of events in presence of a positive SE (reduced right ventricular contractile reserve). With a power of 90% and an alpha error of 5%, a sample size of 238 patients is required with a 3 -year follow-up. The newly recruited patients will add-up to the 116 patients already recruited in years 2018–2020 with the same methodology as a part of the same project in SE2020 [1].

#### 2.12.5. Study Hypothesis

Repaired Tetralogy of Fallot patients with better right (and possibly left) ventricular reserve, and better chronotropic and CFVR will have less chance of developing adverse events in their natural history.

### 2.13. Stress Echo for Surveillance Post-COVID-19 (SECOV)

#### 2.13.1. Background

Cardiovascular abnormalities are observed in half of all COVID-19 patients and may range from RWMA to interstitial lung disease with alveolar capillary distress [91], global contractile dysfunction, coronary microvascular abnormalities [92], and cardiac autonomic dysfunction [93]. In addition, pulmonary hypertension is a recognized risk factor in the short-term [94] and valves are a possible disaster-hit area since myocardium and valve stromal fibroblasts are rich in ACE2 which is the receptor for SARS-CoV-19 [95]. The clinical picture can be complicated by the frequent coexistence of cardiovascular comorbidities.

The common pathogenetic mechanism underlying the spectrum of clinical manifestations and in particular the myocardial involvement following COVID-19 is not fully elucidated; however, a direct cardiac injury could be hypothesized. In some cases, SARS-CoV-2 can cause direct damage to myocytes mediated by stimulation of the angiotensin-converting enzyme 2 (ACE2), which is expressed on myocytes and vascular endothelial cells, acting as a receptor for SARS-CoV-2 and as “gateway” for the virus in these cells. Another hypothesized mechanism is myocardial damage induced by hypoxia and activation of the innate immune response with release of pro-inflammatory cytokines: a real inflammatory storm, also termed cytokine release syndrome, as well as the activation of adaptive auto-immune which can induce vascular and myocardial inflammation and an excess of blood clotting, with consequent episodes of diffuse thrombosis and shock. As a result, patients with COVID-19 pneumonia can rapidly develop severe complications such as respiratory failure, renal failure, or liver dysfunction, which can affect both thromboembolism and bleeding status. This infection is therefore associated with high morbidity and mortality largely due to respiratory failure, with microvascular pulmonary thrombosis perhaps playing an important pathophysiological role, like in other models of viral pneumonia. In previous reports, despite high prevalence of normal cardiac ultrasound, marked elevation of D-dimer, increase of pulmonary artery pressures and RV dysfunction were depicted as common conditions among patients with severe COVID-19 pneumonia and cardiac injury, associated with higher risk of in-hospital mortality [96].

The versatile platform of ABCDE-FG plus L, P, and R SE is ideally suited to identify functional abnormalities, stratify prognosis, and personalize treatment in this complex and expanding population.

#### 2.13.2. Aims

The primary aim is to assess the feasibility of an integrated ABCDEFG (+LPR) approach in post-COVID 19 patients. The secondary aim is to assess the prevalence of abnormalities of different SE parameters in populations stratified according to severity of COVID-19 and clinical variables (age at exposure, cardiovascular risk factors, biomarkers such as troponin, cardiac natriuretic peptides, C reactive protein, lymphocyte count, D-Dimer). The tertiary aim is to assess the prognostic value of individually considered or combined SE indices in prognostic modeling using traditional risk factors and COVID-19 variables.

#### 2.13.3. Methods

The relevant parameters related to COVID-19 infection will be collected and analyzed under the coordination of a cardiologist specialized in COVID-19 treatment. Parameters will include clinical data (days in intensive care, admission length), biomarkers during admission (CRP, BNP, or NT-pro-BNP, troponin), resting transthoracic echocardiography and lung ultrasound at discharge, other imaging data (chest computed tomography), and additional data. SE will be performed from 3 months to 3 years after infection.

#### 2.13.4. Sample Size Calculation

Early experience suggests an incidence of composite outcome end-points defined as in project 1 around 10% per year. If we assume a positivity rate to SE (by composite criteria) of 20%, with doubling of likelihood of events in presence of SE positivity (by any criteria), with a power of 90%, an alpha error of 5%, and an attrition rate of 10%, a sample size of 406 patients is required.

#### 2.13.5. Study Hypothesis

Post-COVID-19 patients have different phenotypes with a spectrum of different underlying functional alterations and therapeutic correlates. SE-ABCDEFG+LPR is useful to identify the pathophysiologic and prognostic heterogeneity underlying similar clinical manifestations allowing targeted therapeutic actions.

### 2.14. RESURGE: Recovery by Stress Echo of Conventionally Unfit Donor Good Hearts

#### 2.14.1. Background

The gold standard and sole curative therapy for advanced stage heart failure is cardiac transplantation. Heart transplantation is limited by severe donor organ shortage. In parallel with population aging, the number of patients listed for transplant steadily increases annually. Paucity of eligible donation severely limits access to cardiac transplantation and leads to increasing wait-list times and avoidable patient mortalities [97]. Two major reasons for exclusion of potential donors is the age > 55 years and concomitance of coronary risk factors. These so called “marginal donors” have high prevalence of occult cardiomyopathy and/or severe CAD which may lead to early primary graft dysfunction and late heart failure even in presence of normal baseline LV ejection fraction before explant [98,99].

The Adonhers (aged donor heart rescue by stress-echo protocol) project was started in 2005 to mitigate the current shortage of donor hearts. In the initial validation phase, hearts with negative SE (step A and step C) were confirmed to have normal or near normal hearts (“too good to die”) at cardio-autoptic verification, whereas hearts with abnormal SE response were invariably associated with severe CAD and/or extensive myocardial scar or necrosis [100]. The ADONHERS study was endorsed and partially funded by the Emilia Romagna Region in the initial Bologna experience and was later scaled up nationwide with endorsement and funding by the Italian Ministry of Health. The project recruited 43 hearts by 2014 and 67 hearts by 2020 from marginal donors discarded according to standard criteria for concomitance of risk factors such as advanced age (>55 years) or multiple risk factors. If the heart showed normal regional and global LV function during stress, then in presence of normal or near normal coronary anatomy the heart was accepted by the cardiac surgeon to donation with favorable short-term [101] and medium-term outcome [102]. With evolving ultrasound technologies and advanced imaging progression in the clinical arena, the assessment of SE can now be safely corroborated with integration of A step with LV quantitative GLS [103] removing the major hurdle of acceptance of SE based on a subjective assessment. These data support the benefit of a SE-driven strategy for donor selection in heart transplantation, since hearts apparently eligible with standard criteria may have occult latent LV dysfunction and hearts apparently non-eligible on the basis of anatomic (minor CAD) findings can be eligible with excellent outcome contributing to mitigate the world-wide problem of heart donor shortage [104,105].

The use of SE would allow to recruit marginal heart donors with normal resting left ventricular function currently dismissed. After a negative SE, at least 50% of these marginal hearts or aged hearts (“silver code hearts”) can be safely rescued to donation. The study will involve all intensive care units with access to cardiology expertise and aims to recruit 500 new hearts in the next 10 years.

#### 2.14.2. Aims

The primary aim is to recruit hearts from donation which are currently excluded by conventional criteria: in particular, aged hearts in patients >55 years and ≤55 years with multiple risk factors. The secondary aim is to assess outcomes in SE-driven transplantation compared to hearts transplanted in the same cardiac surgery centers on the basis of conventional criteria. The tertiary aim is to assess the additional prognostic value, if any, of other signs (B, D, and E) collected in these patients, not used for decision-making and not disclosed to referring physician and used to characterize important aspects of donor heart. These aspects may include diastolic function, preload reserve, coronary microvascular function, and residual innervation of the intrinsic cardiac autonomic system through assessment of HRR in donor heart [106].

#### 2.14.3. Methods

In case of donor with age >55 years or ≤ 55 years but with concomitant ≥3 risk factors (diabetes, hypertension, smoking, obesity, hypercholesterolemia) or history of cardiac arrest, the cardiologist dedicated to the project will reach the hospital in which the potential donor is admitted. The examination of the heart starts with a resting transthoracic echocardiography assessing RWMA (17-segment model of the left ventricle), left ventricular ejection fraction, valve function, diastolic function, and left ventricular hypertrophy according to current guidelines [7,9,107,108]. Exclusion criteria are: resting wall motion score index >1.0; ejection fraction <45%; diastolic dysfunction of grade 2 or more; hemodynamically significant (moderate or higher) valve regurgitation or stenosis; severe left ventricular hypertrophy (left ventricular mass index >175 g/m^2^).

To select the donor, a pharmacological stress echocardiography with dipyridamole (0.84 mg/kg over 6 min) is recommended with the protocol endorsed by guidelines [7,9]. Only in case of contraindications or per hospital specific protocol the second choice will be dobutamine (up to 40 mcg/kg/min for 3′ each step, total maximum infusion time 15 min, without atropine, ineffective in brain dead patients). The diagnostic end-points are stress-induced RWMA and abnormalities in global LVCR. SE will be stopped in case of: hypertensive pressure response (systolic blood pressure >220 mmHg, diastolic blood pressure >120 mmHg), absolute or relative hypotension (reduction of blood pressure >30 mmHg), supraventricular arrhythmias (supraventricular tachycardia or atrial fibrillation), ventricular arrhythmias (ventricular tachycardia, frequent ventricular premature beats). A maximal stress would be required, that is a positive ischemic response at any stage or a negative response after the full drug dose, because a submaximal stress test could have a limited value in ruling out significant coronary or myocardial disease [109]. Great care will be taken to maintain hemodynamic stability of the potential donor to avoid damage of the other organs.

All images will be recorded, stored, and analyzed as per guidelines and similarly to the other projects [7,9], with special emphasis on wall motion score index and LVCR based on ejection fraction and force (average of three consecutive cycles).

#### 2.14.4. Data Analysis

Data analysis will include 3 different steps: 1—Mandatory: rest and peak stress evaluation of wall motion score index. Potential donors will be excluded in presence of any resting or stress-induced RWMA, corresponding to a resting or peak stress wall motion score index >1.0; 2—Mandatory: increase in LVCR assessed with ejection fraction and force. It requires an accurate assessment of left ventricular volumes and must be obtained in each and every patient. If data regarding left ventricular volumes are not available or not considered reliable, evaluation cannot be started. Potential donors with any decrease of ejection fraction or force during stress will be excluded from donation (ejection fraction stress < rest or force stress < rest); 3—Optional, important but not mandatory: variation of CFVR in left anterior descending coronary artery. This information will be obtained in around 50% of patients. However, if information on CFVR is missing, this should not stop donor’s enrollment since this information is of investigational interest at present without a clear outcome correlated in this very peculiar setting.

#### 2.14.5. Management of Donors after SE

The potential donors with abnormal baseline or SE will be excluded from the transplant. In the case of normal baseline echocardiography according to previous reported criteria, the donor will be eligible for donation if SE shows normal regional wall motion and normal global LVCR. SE provides complementary functional data and cannot replace anatomic information of coronary angiography. The final acceptance of the heart follows the clinical and emergency criteria, as indicated by internationally accepted UNOS criteria. Data regarding CFVR will be recorded as additional, optional data not directly affecting decision-making. After the cardiac transplant, the recipients according to routine management will undergo invasive coronary angiography and left ventriculography at 1 month and, thereafter, once a year.

The hearts excluded from donation for RWMA or abnormal LVCR could however be collected for heart valve preparation and evaluated by coronary angiography and by pathological examination according to local facilities. In particular, RWMA are usually associated to severe CAD in the related myocardial territories and the absence of incremental value of pressure/LV end-systolic volume ratio during stress correlated to LV damage (mostly acute due to catecholaminergic surge/ischemic response during brain death or, less frequently, chronic fibrotic scar).

#### 2.14.6. Sample Size Calculation

The primary end-point of the study is all-cause death through 10 years (5-year accrual period for recruitment plus additional 5-year follow-up). According to the most recent data of the registry of the International Society of Heart and Lung Transplantation showing a death rate of 27.5% in the standard group over a similar follow-up period and considering a similar follow-up period, an overall sample size of 800 patients allocated 1:1 (n = 400 per group) to the age-matched standard selection group and to patients selected according to SE-driven criteria will achieve 90% power at a 0.050 significance level to detect an equivalence hazard ratio of 1.25 when the actual hazard ratio is an equivalence hazard ratio of 1.00 assuming no patient drop-out during the follow-up period.

### 2.15. SEMIR-Stress Echo in Ischemic Mitral Regurgitation

#### 2.15.1. Background

Chronic ischemic mitral regurgitation is a frequent complication of CAD, and is associated with a poor prognosis and outcome. After myocardial infarction ischemic mitral regurgitation is associated with doubling of mortality rates [110,111]. The role of concomitant mitral valve surgery for ischemic mitral regurgitation in patients undergoing coronary artery bypass grafting remains controversial [112]. Furthermore, there is no consensus on the cut-off value of ischemic mitral regurgitation. The current European Society of Cardiology guidelines [113] considers secondary mitral regurgitation as severe in patients with effective regurgitant orifice >0.2 cm^2^, regurgitant volume >30 mL. However, in the 2017 focused update on Management of Valvular Heart Disease [114], the definition of severe secondary mitral regurgitation is now the same as for severe primary mitral regurgitation (effective regurgitant orifice area ≥0.4 cm^2^, regurgitant volume ≥60 mL, regurgitant fraction ≥50%). It also underlines the gaps in evidence on the potential impact of mitral valve intervention on survival of patients undergoing coronary artery bypass grafting with effective regurgitant orifice 0.2–0.39 cm^2^, and regurgitant volume 30–59 mL. Recognizing the dynamic nature of ischemic mitral regurgitation, SE could clarify the indications for concomitant mitral valve surgery during coronary artery bypass grafting.

#### 2.15.2. Aims

To assess the value of SE testing as an indicator of outcome in patients with resting moderate mitral regurgitation (effective regurgitant orifice 0.2–0.39 cm^2^, and regurgitant volume 30–59 mL) of ischemic origin.

#### 2.15.3. Methods

All patients (“all-comers”) with angiographically documented CAD, moderate ischemic mitral regurgitation (effective regurgitant orifice 0.2–0.39 cm^2^, and regurgitant volume 30–59 mL) will be included in the study. ABCEFG (+LPR) SE will be performed on all the patients. All patients will enter a regular clinical follow-up program with annotation of cardiovascular and non-cardiovascular endpoints as specified in project 1. Patients undergoing bypass surgery with or without mitral repair or percutaneous coronary angioplasty with or without mitral valve intervention will be separately analyzed.

#### 2.15.4. Sample Size Calculation

The expected incidence of composite end-points as defined in project 1 is around 20% per year in patients with increasing ischemic mitral regurgitation [115] left on medical therapy without valve repair. Considering a 5-year follow-up and knowing that expected incidence of mitral regurgitation increasing is around 25% [115], with a power of 90% and an alpha error of 5%, a sample size of 173 patients per arm (bypass or angioplasty) is required.

#### 2.15.5. Study Hypotheses

The primary study hypothesis is that patients with moderate (grade 2 and 3, American Society of Echocardiography 2017) mitral regurgitation worsening of ≥1 grade during exercise (effective regurgitant orifice ≥0.4 cm^2^, regurgitant volume ≥60 mL, regurgitant fraction >50%) have a worse outcome on medical therapy and greater benefit from mitral valve correction than patients with no change or improvement with SE. The secondary study hypothesis is that patients with lower LVCR (by EF or force criteria), more B-lines during stress, higher systolic pulmonary artery pressure, lower pulmonary vascular reserve, and higher LAV dilation during SE (pre-surgery) will have worse prognosis independent of mitral regurgitation severity and with either treatment (medical therapy or valve repair).

### 2.16. SEVA: Stress Echocardiography in Valvular Heart Disease

#### 2.16.1. Background

SE is recommended in valvular heart disease for three main categories of applications characterized by a mismatch between resting transthoracic echocardiography findings and symptoms during exercise or activities of daily living: 1. Severe valve disease without symptoms; 2. Non-severe single- or multi-valve disease with symptoms; and 3. Symptomatic valve disease of indeterminate severity in context of low flow. In all of these conditions, SE may provide unique information to match symptoms with degree of cardiac involvement, for risk stratification and to guide decision-making, determining the optimal timing for surgery or percutaneous interventions [115].

Despite the enormous potential information to be gained, SE lacks supportive evidence in this particular field. Most recommendations are based on classes of evidence C (“consensus opinion of experts and/or small, retrospective studies”). Existing recommendations emphasize idealized cut-offs such as stress-induced increase in ejection fraction >4% in aortic regurgitation, systolic pulmonary artery pressure values >60 mm Hg with stress in mitral stenosis or mitral insufficiency or valve area <1.0 cm^2^ in aortic stenosis [16]. The evidence supporting these simple cutoffs are not exactly written in stone, and they are vulnerable to artifacts, with limited reproducibility, known sources of error and inadequate validation [116]. Some of these applications are not so easy to execute and may not be safe if performed outside of a dedicated and experienced SE laboratory, and yet the recommended caseload for a level III echo competencies include 200 SE studies per year of which 25 need to be non-coronary indications [117], meaning that a laboratory can perform only 1 or 2 SE studies per year in low-flow low-gradient aortic stenosis and remains competent. As a consequence, SE in valvular heart disease is losing ground in evidence-based guidelines and practice. For instance, SE in low-flow low gradient aortic stenosis is the most frequent application of SE in valvular heart disease but it was “taken out” from the European Society of Cardiology guidelines on valvular heart disease in 2017 [112]. At this time, we need stronger evidences that are based on comprehensive evaluation of patients with valvular heart disease, since inducible ischemia, pulmonary congestion, alterations of preload and contractile reserve, coronary microvascular dysfunction and dysregulation of cardiac autonomic function, left and right atrial volume changes, abnormal pulmonary hemodynamic response, and limited right ventricular function reserve in response to stress can be even more important than the valve condition itself in determining the outcome of patients with valvular heart disease. The pathophysiological characterization of disease complexity is the prerequisite for a targeted therapeutic approach.

#### 2.16.2. Aims

The primary aim is to evaluate the feasibility of ABCDEFG-SE plus L (left atrium), P (pulmonary vascular reserve), and R (right ventricular function) in these patients. The secondary aim is to assess the correlation of each SE parameter with indices of functional severity (New York Heart Association Class, cardiac natriuretic peptides, peak oxygen consumption, etc.). The tertiary aim is to assess the prognostic value of SE for prognostic stratification in the long-term.

#### 2.16.3. Methods

Patients referred to SE lab for valvular heart disease will be enrolled and studied with semi-supine bicycle SE, with the exception of low flow low gradient aortic stenosis in which pharmacological test with low dose dobutamine is preferred. Seven distinct groups will be recruited: 1—asymptomatic severe aortic stenosis; 2—Low-flow, low-gradient severe aortic stenosis with reduced ejection fraction; 3—asymptomatic severe or symptomatic non-severe primary mitral insufficiency; 4—“asymptomatic” severe or symptomatic non-severe mitral stenosis; 5—“asymptomatic” severe or symptomatic non-severe aortic regurgitation; 6—asymptomatic or symptomatic non-severe multivalvular disease; 7—post heart valve procedures (prostheses and valvuloplasty).

#### 2.16.4. Sample Size Calculation

Expected incidence of SE positivity (by composite criteria) is around 50% in these patients, ranging from 20% positivity of Step B to 40% of Step C and 35% of Step D [118,119]. Considering the Cox proportional hazard model for the prognostic analysis (tertiary end-point), if we conservatively assume a 20% incidence of composite end-points (as defined in project 1) at 3 years and a doubling of events occurrence in the presence of a positive SE (for at least one extra-valvular criteria), then a sample size of about 217 patients per sub-group is required to evaluate the tertiary endpoint with 90% power and an alpha error of 5%. An exception to this estimation applies to the sub-group of patients with low-flow low-gradient aortic stenosis, in whom a sample size of 154 patients is required with a 2-year follow-up is sufficient, due to the higher incidence of composite end-points [120].

#### 2.16.5. Study Hypothesis

In patients with valvular heart disease meeting the inclusion/exclusion criteria of an appropriate indication for SE, those with less inducible ischemia, less pulmonary congestion, higher LV preload and contractile reserve, better coronary microcirculatory and cardiac sympathetic reserve, lower pulmonary hemodynamic resistances and better right ventricular functional reserve will have a more favorable outcome than patients with the same degree of valvular stenosis or regurgitation but different overall vulnerability.

### 2.17. SESPASM—SE for Coronary Vasospasm

#### 2.17.1. Background

Coronary artery spasm is considered one of the major mechanisms causing dynamic stenosis of epicardial coronary arteries, which can evoke acute myocardial ischemia, variant angina. unstable angina, acute myocardial infarction, syncope, and sudden death [121]. In microvascular angina, the same pathophysiologic substrate, microvascular spasm, and impaired dilatation, cause the anginal pain, decrease quality of life and carry adverse prognosis. Its clinical recognition is still elusive and challenging [121]. Classically, coronary artery spasm is diagnosed by a provocative intracoronary ergonovine injection during diagnostic coronary angiography [121] but the test is seldom used in the real world since it requires hours of catheterization laboratory occupancy, lack of specific reimbursement, and it is risky due to invasiveness, with high cumulative radiation exposure and relatively large doses of nephrotoxic iodine contrast injections required for serial coronary artery imaging. Moreover, provocative invasive testing with acetylcholine for microvascular spasm is solely based on absence of epicardial spasm, ECG changes or angina pain, because microvascular network could not be seen. The lack of a suitable noninvasive test has limited the applicability of noninvasive selective testing for coronary vasospasm, but early experiences in the eighties [122] and contemporary large-scale experiences from multicenter studies in over 14,000 patients have conclusively shown that testing for vasospasm is highly accurate and extraordinarily safe outside the catheterization laboratory in appropriately selected patients [123]. The recognition of coronary vasospasm is also clinically important since standard therapy of angina such as percutaneous coronary interventions or beta-blockers can be ineffective or even worsen symptoms, whereas the prognosis is relatively benign when vasospasm is recognized and the patient is treated with calcium channel blockers and nitrates. Coronary vasospasm can be the unrecognized cause of other life-threatening conditions such as unexplained syncope or unexplained resuscitated cardiac arrest [123,124,125,126,127]. Regarding the specific test to be used, ergometrine is highly effective but not commercially available in most countries. A similar sensitivity can be obtained with hyperventilation [128] combined (if negative at 5′ after testing) with exercise [129]. This allows to have a strong stressor avoiding pharmacological testing and using hyperventilation and exercise testing [130] both recommended by existing guidelines for the noninvasive work-up of patients with suspected coronary vasospasm. These two steps give us diagnostic opportunity to assume whether angina is caused by epicardial spasm (obvious RWMA, A) or by coronary microvascular dysfunction (limited increase in flow, D). Large scale validation of safety and efficacy of noninvasive testing of coronary vasospasm is however missing to date. This project fits well with the future directions recently identified by international experts suggesting the establishment of an international coronary vasomotor disorder clinical registry for diagnostic, prognostic, and therapeutic research [130].

#### 2.17.2. Aims

The primary aim is to evaluate the feasibility and safety of hyperventilation and exercise ABCDE-SE in appropriately selected patients with angiographically normal coronary arteries and an intermediate-to-high pre-test probability of coronary vasospasm of epicardial arteries or microvasculature. The secondary aim is to assess the positivity rate of A and D criteria in these patients, compared to standard ECG criteria. The tertiary aim is to assess the prognostic value of the different responses of SE leading to SE-driven therapies.

#### 2.17.3. Methods

Only patients with strong (Class 1) indication to vasospasm testing according to the recent guidelines and recommendations (Japanese Coronary Spasm Association 2014, COVADIS group 2018) will be initially considered [121,131,132]. The most appropriate indications consist of 5 groups in patients with angiographically normal coronary arteries: 1-chest pain with at least one clinical suspicion criteria. They are: a—chest pain at rest and/or at night and/or early morning; b—marked circadian variability in exercise tolerance; c—history of angina precipitated by hyperventilation; d—calcium channel blockers, but not beta-blockers, suppress angina, or angina is associated with the use of drugs known to induce coronary vasospasm in vulnerable patients, such as ergometrine given in the obstetric clinic to reduce uterine blood loss in the puerperium phase or bromocriptine given for milk suppression, sumatriptan, or ergometrine used in neurology for migraine headaches, fluorouracil and capecitabine (an oral 5-fluorouracil prodrug) given as chemotherapy in breast and colon-rectal cancer, and, with increasing frequency, cocaine as a cause of chest pain; 2-Recurrent chest pain with documented myocardial ischemia by ECG following successful percutaneous coronary revascularization (with patent dilated arteries at angiographic verification prior to vasospasm testing). 3-Unexplained resuscitated cardiac arrest. 4-Unexplained syncope with antecedent chest pain; 5-Previous myocardial infarction (MINOCA); 6-Tako-Tsubo syndrome.

Exclusion criteria are: previous documentation of transient ST segment elevation ≥0.1 mV or depression ≥0.1 mV or appearance of new negative U waves by Holter or 12-lead ECG during spontaneous chest pain; positivity at SE with exercise, dobutamine or vasodilators, with recognition of typical vasospastic positivity during or at recovery of exercise [133]; during infusion or following antidote administration with dobutamine [134]; during infusion or following aminophylline administration with dipyridamole [135]; coronary vasospasm during invasive testing; or resting global LV dysfunction (Wall Motion Score Index >1.4 or ejection fraction <40%).

Since vasospasm susceptibility peaks in early morning, patients prepared for exercise testing will undergo vasospasm testing in the morning (between 8 and 11 a.m.) with hyperventilation (deep and frequent breaths, at least 25 up to 35 per minute, for 5 min followed by additional 5 min monitoring). If negative or equivocal at 5 min after the end of hyperventilation, the patient will start exercise with the usual protocol. Discontinuation of therapy with nitrates and calcium-antagonists (not beta-blockers) for at least 24 h before testing is recommended. Preliminary experience gained in Belgrade within SE2020 network showed the feasibility of this approach with higher ischemic power than hyperventilation alone [136].

#### 2.17.4. Sample Size Calculation

The expected incidence of SE positivity (by at least one of the ABCDE criteria) is around 35% [136]. For tertiary composite outcome end-points of the study defined as in project 1, considering the Cox proportional hazard model to assess prognostic relevance of SE, if we conservatively assume a 5% yearly incidence with doubling of likelihood of events in presence of a positive SE (for RWMA criteria), with a power of 90% and an alpha error of 5%, a sample size of 513 patients is required with a 5-year follow-up for the composite endpoint. The estimated sample size for the tertiary endpoint will also be enough to evaluate primary and secondary endpoints.

#### 2.17.5. Study Hypothesis

SE for coronary vasospasm (with single stress hyperventilation or two-stresses hyperventilation + exercise) is highly safe and feasible with excellent success rate and allows to identify a potentially benign condition (if recognized and treated) such as coronary vasospasm in a significant number of patients otherwise missed by conventional invasive and noninvasive approaches.

## 3. Conclusions

SE has many unique peculiarities that make it especially attractive in the current era for the possibility to minimize the global legal, economic, societal, and environmental burden due to cardiac imaging. In fact, SE is physician-friendly, patient-friendly, payer friendly, and planet friendly (Table 2).

SE2020 was the reading and writing classes of a new diagnostic alphabet. Everyone can now read and write the new simple ABCDE alphabet, with each letter providing independent and incremental value in predicting survival [137]. With SE2030 [138], all readers will read and write also F, G, and occasionally L, P, and R (Table 3). Beyond A, there is a whole new alphabet and we need them all to open the door of personalized medicine to SE.

## Figures and Tables

**Table 1:** General inclusion/exclusion criteria.

	Inclusion Criteria	Exclusion Criteria
Age > 18 years *	√	
Acceptable acoustic window at rest (≥14 out of 17 LV segments)	√	
Clinically indicated test	√	
Informed consent	√	
Acute coronary syndromes or acute heart failure		√
Acute pulmonary embolism, myocarditis, pericarditis, or aortic dissection		√
Serious cardiac arrhythmias (ventricular tachycardia, complete atrio-ventricular block)		√
Prognosis-limiting (survival <1 year) extra-cardiac disease		√
Resting systolic blood pressure >180 mmHg or significant hypotension		√

* Except for project 7 that may include younger patients with repaired tetralogy of Fallot after parental consent.

**Table 2:** The four main features of stress echo in the era of sustainability.

Legal sustainability	Non-ionizing radiation	Patient-friendly
Clinical sustainability	Versatile (for all pts)	Physician-friendly
Economic sustainability	Low cost, high value	Payer-friendly
Environmental	Low carbon emission	Planet-friendly

**Table 3:** ABCDE-FGLPR Steps, specific parameters, target patients.

Steps	Key Parameters	Patients
Step A	RWMA	All (mostly CAD + HF)
Step B	B-lines	All
Step C	EDV, ESV	All
Step D	CFVR-LAD	All
Step E	HRR	All
Step F	MR severity	CAD + HF + VHD + HCM
Step G	LVOTO	CAD + HF + VHD
Step L	LA volume	CAD? HFpEF? HCM? VHD?
Step P	E/e’, TRV or ACT	CAD? HFpEF? HCM? VHD?
Step R	TAPSE/PASP	CHD, VHD?

## Data Availability

The data presented in this study are available on request from the corresponding author. The data are not publicly available due to privacy law.

## Abbreviations

The following abbreviations are used in this manuscript:

AI	artificial intelligence CAD, coronary artery disease
CFVR	coronary flow velocity reserve
GLS	global longitudinal strain
HCM	hypertrophic cardiomyopathy
HRR	heart rate reserve
LV	left ventricle
LVCR	left ventricular contractile reserve
RWMA	regional wall motion abnormality
SE	stress echocardiography
